# Fine-scale spatial variability of heat-related mortality in Philadelphia County, USA, from 1983-2008: a case-series analysis

**DOI:** 10.1186/1476-069X-11-16

**Published:** 2012-03-25

**Authors:** David M Hondula, Robert E Davis, Matthew J Leisten, Michael V Saha, Lindsay M Veazey, Carleigh R Wegner

**Affiliations:** 1 Department of Environmental Sciences, University of Virginia, Charlottesville, USA

**Keywords:** Biometeorology, Heat waves, Climatology, Apparent temperature, Spatial analysis, Heat-health impacts, Remote sensing, Landsat

## Abstract

**Background:**

High temperature and humidity conditions are associated with short-term elevations in the mortality rate in many United States cities. Previous research has quantified this relationship in an aggregate manner over large metropolitan areas, but within these areas the response may differ based on local-scale variability in climate, population characteristics, and socio-economic factors.

**Methods:**

We compared the mortality response for 48 Zip Code Tabulation Areas (ZCTAs) comprising Philadelphia County, PA to determine if certain areas are associated with elevated risk during high heat stress conditions. A randomization test was used to identify mortality exceedances for various apparent temperature thresholds at both the city and local scale. We then sought to identify the environmental, demographic, and social factors associated with high-risk areas via principal components regression.

**Results:**

Citywide mortality increases by 9.3% on days following those with apparent temperatures over 34°C observed at 7:00 p.m. local time. During these conditions, elevated mortality rates were found for 10 of the 48 ZCTAs concentrated in the west-central portion of the County. Factors related to high heat mortality risk included proximity to locally high surface temperatures, low socioeconomic status, high density residential zoning, and age.

**Conclusions:**

Within the larger Philadelphia metropolitan area, there exists statistically significant fine-scale spatial variability in the mortality response to high apparent temperatures. Future heat warning systems and mitigation and intervention measures could target these high risk areas to reduce the burden of extreme weather on summertime morbidity and mortality.

## Background

High heat and humidity pose a major health threat for residents of middle latitude climates during the warm season. In the United States, for example, heat ranks as the leading cause of weather-related mortality. Many of these deaths are believed to be preventable via the implementation of appropriate mitigation measures such as advanced notification of at-risk groups and availability of cooling shelters [1]. Research to date has examined the aggregate heat-health response for large metropolitan areas and identified robust relationships for many locales. When temperature-humidity measures rise above a geographically sensitive threshold, human mortality becomes greater than the typical seasonal pattern would suggest [2]. The consistency of this heat-mortality relationship has spawned heat warning systems across the globe for forecast zones comprising entire metropolitan areas or multiple counties [1]. Within these metropolitan areas, however, there exists considerable variability in environmental conditions and demographic and social characteristics of the population. Here we explore the relationship between high heat and humidity and human mortality at the local scale. Past work has primarily focused on a larger scale by examining the response of an entire metropolitan area, but the allocation of resources intended to protect citizens from the dangerous effects of heat and humidity could be improved with more specific knowledge of where the risk is highest within urban areas. The current state of reporting and data availability makes it possible to assess this risk with a multidecadal record of geographically-specific observations. In this manuscript we utilize such a record to evaluate intra-city mortality risk within a major United States metropolitan area.

Elevated temperatures are commonly observed in city centers when compared to surrounding areas because of the well-documented urban heat island (UHI) effect [3]. Higher temperatures are observed in areas with tall buildings, high building density, limited green space, industrial land use, and anthropogenic heat sources [4]. The complex nature of cities leads to large differences in temperatures between varying neighborhoods--differences that may lead to certain areas exceeding physiological thresholds related to heat stress while other locations maintain thermal comfort [5].

Demographic and social variability within metropolitan areas may also contribute to geographic variability in heat-related mortality risk. Age is the most commonly-cited demographic factor related to morbidity and mortality risk during heat events [6,7]. Elderly populations are believed to be at higher risk to changes in temperature than the general population because of diminished or compromised thermoregulatory capacity [1].

The impact of race on heat-related mortality is not as clear. Minority populations have often been linked to elevated mortality during heat events [8-10], but other research found no significant mortality difference between races [11,12]. Socioeconomic status is potentially linked to heat-related mortality because affluent residents may be more able to afford higher-quality housing and air conditioning [13]. Areas with higher poverty rates were associated with statistically higher mortality rates in the 1993 Philadelphia heat event [10]. A wide range of other factors have also been linked with, or suggested to be linked with, elevated mortality rates during heat events, including education level, gender, aerobic fitness, activity level, and pre-existing medical conditions [7].

Over roughly the past decade, researchers have started examining the spatial distribution of heat-related mortality within metropolitan areas. At the local scale, places with higher morbidity and mortality rates during heat events have been associated with lower neighborhood stability and income levels [10,13] and proximity to the downtown core [14-16]. In addition, remote sensing imagery has been incorporated and places with higher thermal readings have also been linked with higher mortality rates [17,18].

The present study aims to relate a multi-decade record of localized mortality data to a large suite of independent variables including demographic, social, and environmental components. Our goal is to determine if significant spatial variability in heat-mortality exists within Philadelphia County, PA, and if so, examine the underlying factors that may be responsible for that variability.

## Methods

### Data

Individual all-cause mortality records for Philadelphia County residents were obtained from the Pennsylvania Department of Health for the period 1983-2008. We limited our analysis to deaths that occurred within the County boundary. The dataset includes the zip code of residence and age of the decedent. Excluding cases where the zip code was not available, the record contains 409,554 deaths over the 26-year period The mortality records provided from the Bureau of Health Statistics and Research within the Pennsylvania Department of Health were de-identified prior to receipt by the researchers (the files do not contain information that could make it possible to identify an individual). The records are maintained and available for approved research purposes. As such, under Title 45 Part 46 exemption category 4, IRB approval was not required for this study.

**Figure 1:**
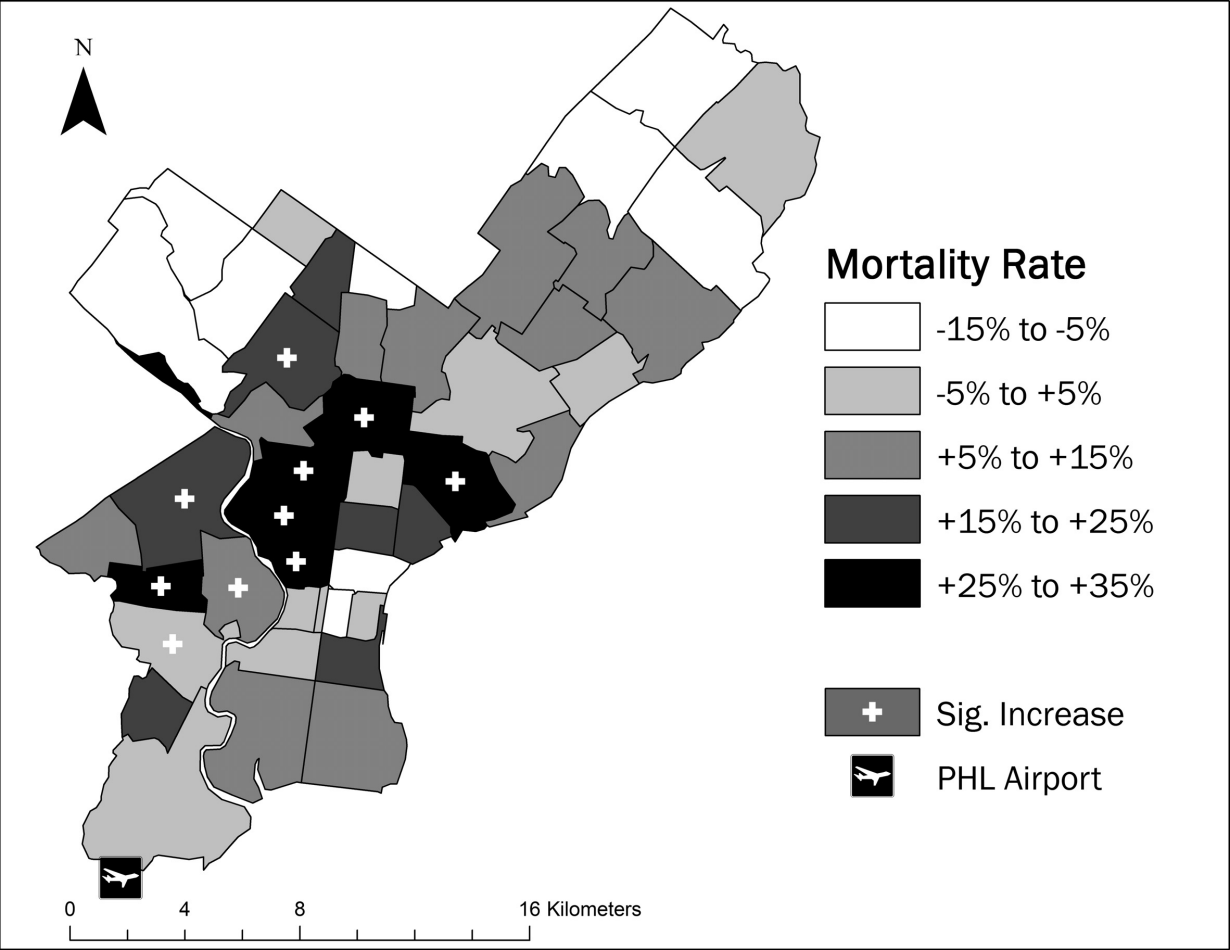
**Extreme heat mortality exceedance rates in Philadelphia County, PA**. Mortality exceedance rates for Philadelphia county ZCTAs on days following those with 7:00 p.m. local time apparent temperatures greater than 34°C. ZCTAs with mortality rates significantly greater than the background rate are identified by a white cross. The location of Philadelphia International Airport (PHL), the source of meteorological data employed in this study, is represented by the airplane symbol.

Surface hourly meteorological data for the entire period of record were obtained from Philadelphia International Airport (see Figure 1), including measurements of air temperature, dew point temperature, and wind speed. We calculated hourly apparent temperature (AT, °C) using a parameterization of the Steadman model

$$\text{AT}=-2.653+(0.994*\text{T})+[0.0153*{\text{T}}_{\text{D}}{}^{2})]+\text{C}$$

where T is the dry-bulb temperature (°C), T_D _is the dew point temperature (°C), and C is a correction based on wind speed (m/s) [9,19,20]. We linearly interpolated the temperature correction for each integer value of wind speed between 0 and 16 m/s because the table of corrections only provides values for coarse increments of wind speed [19]. The correction for 16 m/s was used in cases where the wind speed exceeded 16 m/s. The airport AT measurements serve as the basis for the identification of days associated with exceptionally high heat and humidity conditions across Philadelphia County.

The suite of variables we incorporated to compare to the spatial pattern in heat-related mortality includes demographic and social factors as well as characteristics of the buildings and land surface. Population counts by age were obtained for each census tract in the County for 1980, 1990, and 2000 from the United States Census Bureau and the National Historical Geographic Information System (NHGIS). Additional variables obtained from the census include year 2000 tract-level measures of race, education level, income, occupancy, and building age (see Table 1). For geographic analysis, we used boundary shapefiles for the census tracts and year-2000 Zip Code Tabulation Areas (ZCTAs, Figure 1) from the Census Bureau and NHGIS.

**Table 1:** List of explanatory variables for assessing the spatial distribution of heat-related mortality exceedances and loadings for extracted principal components

	**Component (% Variance Explained)**					
	
	1 (35.9)*	**2 (16.4)**	**3 (10.2)**	**4 (7.7)**	5 (5.5)*	6 (4.4)*
	
ZONING AND LAND USE						
	
% Low Density Residential	**-.636**	-.290	.122	.398	.017	.088
	
% Mid Density Residential	-.402	-.456	.012	-.015	-.578	.087
	
% High Density Residential	**.702**	.131	-.217	-.079	.449	-.129
	
% Recreational	-.404	-.438	-.058	.304	.396	-.323
	
% Industrial	.161	.020	**.646**	-.420	.229	.161
	
% Mixed Use	-.073	.142	-.256	.394	.434	.592
	
% Commercial	.045	**.797**	-.418	.015	.029	.036
	
% Building Coverage	.514	**.663**	-.442	-.053	-.049	-.078
	
DEMOGRAPHICS						
	
% White	**-.647**	**.610**	.361	-.129	.046	-.060
	
% Black	.471	**-.648**	-.513	.086	-.071	.051
	
% American Indian	**.655**	.039	.457	.287	.012	-.028
	
% Asian	-.017	**.695**	-.165	.144	-.244	.099
	
% Pacific Islander	.400	.271	.255	**.656**	.003	.181
	
% Other race	**.615**	.146	.541	.333	-.079	-.041
	
% Two or more races	.500	.324	.292	.572	-.334	-.110
	
% Nonwhite	**.647**	**-.610**	-.361	.129	-.046	.060
	
% Over age 65	-.345	.300	-.003	.142	.159	**-.620**
	
% Without hs diploma	**.799**	-.135	.402	-.191	.107	-.095
	
Median per capita income	**-.651**	.455	-.328	.181	.078	-.103
	
% Below Poverty Line	**.912**	-.005	.006	.066	.266	.050
	
% Below 2x Poverty Line	**.925**	-.115	.060	-.053	.202	-.033
	
% Living Alone over age 65	**-.648**	.044	.370	-.187	.095	.177
	
% Living Alone	**-.768**	.260	.086	-.085	.200	.247
	
SURFACE TEMPERATURE						
	
Surface Temp. Image (5/15/2004)	**.762**	.360	.001	-.332	-.201	.107
	
Surface Temp. Image (7/29/2008)	**.849**	.340	-.152	-.261	-.124	.042

Loadings greater than 0.6 or less than -0.6 are shown with bold text and statistically significant components in the regression model are identified with an asterisk

We used zoning maps to further assess the surface characteristics of the built environment. Zoning ordinances constrain the use, coverage, form, and spatial arrangement of urban development. These regulations can have significant effects on urban environments [21]. Thus, the zoning variables may serve as proxies for high-resolution thermal measurements given that air temperature sensors are not available at the same level of spatial detail. Building density and zoning information for the County were obtained from the Pennsylvania Spatial Data Access (PASDA) clearinghouse GIS database. Both the zoning and building files contain thousands of individual polygons identifying each of several dozen zoning categories and individual building elements.

**Figure 2:**
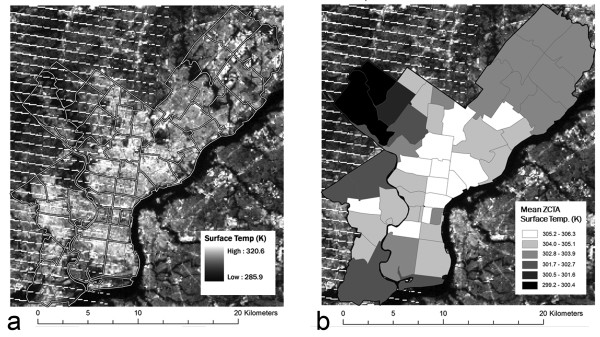
**Thermal imagery of the Philadelphia metropolitan area**. (a) Landsat thermal image of surface temperatures (K) in Philadelphia County, PA, from July 29, 2008. Zip code tabulation areas (ZCTAs) that comprise the county are shown with white outlines. (b) The mean surface temperature within each of the Philadelphia County ZCTAs is shown with grayshading.

To assess intra-city variability in thermal stress, we utilized imagery of the Philadelphia area from the Landsat 7 Enhanced Thematic Mapper Plus (ETM+). Landsat is a sun-synchronous satellite with a 16-day overpass interval. We downloaded 47 warm-season relatively-cloud free images using the USGS Global Visualization Viewer "Glovis" spanning the period 2004-2010 and selected two images that corresponded with periods of extremely elevated air temperature measured at Philadelphia International Airport (e.g., Figure 2).

### Modeling

To obtain fine-scale mortality counts, daily all-cause mortality is aggregated for each of the 48 ZCTAs comprising Philadelphia County for the periods 1983-2008 (9,490 days). These data are de-seasoned to remove any residual effects of the intra-annual mortality cycle, and age-standardized to account for temporal changes in population demographics. All-cause data are commonly employed in heat impact research because of the lack of a standardized definition for heat-related death and the potential for heat stress to contribute to other causes of mortality (especially respiratory and cardiovascular causes). We calculated the seasonality as the 30-day LOWESS-smoothed daily means of the County-wide mortality sum. We then scaled this seasonality model such that the mean of the seasonality curve for each ZCTA-year matched the background mortality rate observed for each ZCTA-year. We next age-standardized the mortality data based on the ZCTA-level population age structure obtained from the 1980, 1990, and 2000 census data using ten age classes (0-4, 5-14, 15-24,...,85 and above) and interpolated (by age class) within each ZCTA.

The de-seasoned, age-standardized daily mortality departures were sorted into AT groups based on the hourly airport data to examine the heat-mortality relationship. After testing different combinations, we ultimately chose to use overlapping 3°C-wide AT bins with a 1.5°C interval between the midpoints of each bin. This particular bin size was chosen based on sensitivity analyses in an effort to balance threshold AT specificity and sample size within each bin. We then calculated the mean mortality departure within each bin. A one-tailed randomization procedure was used to identify a significant response for a particular AT bin. The mean excess mortality for a given AT range is compared to the 95^th ^percentile mean of 10,000 randomly-drawn subsets of the same sample size as the test group. Samples were drawn exclusively from days falling within the warm season, defined here as between calendar days 150-275 (approximately June-September). If the observed mortality is above the 95^th ^percentile, we identify a statistically significant mortality elevation. We excluded any bins with a sample size of 5 or fewer cases from analysis. The randomization procedure is used in place of a traditional *t*-test because of the non-normal distribution of the daily mortality departures [22]. In all cases, the mortality response is expressed as a percent difference relative to the mean warm-season mortality in Philadelphia County (or within each ZCTA) of 0.26 deaths per 10,000 residents per day.

We first used the randomization procedure to evaluate the mortality response by AT for the entire County (total daily sum). We tested the mortality response by AT and hour for 12:00 a.m. to 8:00 p.m. on the day of death and all 24 hours for the two days immediately prior (each of 68 hours was tested for each AT bin). (We did not examine AT impacts after 8:00 p.m. local time on the day of death.) Examination of the overall city response was used to guide the local-scale analysis to an AT/time combination when the mortality signal was robust. The minimum value of the first bin above which mortality remains consistently significantly greater than zero was adopted as the threshold AT for the given hour [2]. We then calculated the overall mortality response when AT values in excess of the threshold were observed (instead of within each AT bin) for the entire County and for each of the 48 ZCTAs. The ultimate dependent variable in the regression model is the mean excess mortality for certain ATs at a given hour of the day and lag. The randomization test was again used to identify significantly elevated mortality at both the County-wide and ZCTA scale, except in this instance the test statistic was based on the cumulative response above the threshold AT instead of within a particular AT bin.

Because the mortality data were provided with zip code of residence, ZCTAs serve as the geographic unit of analysis. All explanatory variables were projected into year 2000 ZCTAs using the Hawth's Tools Polygon-In-Polygon feature within ESRI ArcMap version 9.3.

The zoning code for Philadelphia includes many different classification types, including over twenty categories of residential zoning alone. Because a large number of these zoning categories were constrained to only a handful of parcels throughout the city, we combined similar zoning categories into seven overall classifications (Table 1). This aggregation was aided by numerical information within the zoning code related to lot size, building heights, etc., as well as street-level photography of the structures present for each zoning type.

Landsat imagery was employed to assess local-scale variability in surface temperatures. We converted the pixel-by-pixel brightness numbers to spectral radiance and then to temperature using the ArcMap raster calculator (Figure 2a) [23]. Once all images were converted to temperature values, we used the ArcMap Zonal Statistics tool to calculate the mean surface temperature within each ZCTA (Figure 2b). We used a mid-morning image from May 15, 2004 (Air temperature = 295.4 K at time of image) and a mid-morning image from July 29, 2008 (300.9 K). These two days best met our criteria of having little or no cloud cover and high air temperature out of the 47 images we downloaded. We added the mean surface temperature by zip code for each image as separate variables into the overall pool.

Principal components analysis was then used to reduce the number of variables from the independent pool and eliminate colinearity. As there are only 48 "cases" (ZCTAs) in the study, reducing the number of independent variables is especially important to avoid over-fitting the regression model.

We used multiple linear regression (conducted in SPSS statistical software, version 19.0.0) to relate the principal components of our independent variables to the local-scale mortality response. Significant variables were deemed to be those with a partial p-value of less than 0.05. The residuals from the regression models were examined for spatial autocorrelation using Moran's *I *statistic in ArcMap to determine if an additional term is needed in the regression model to properly account for the true degrees of freedom in a spatially autocorrelated field.

## Results

**Figure 3:**
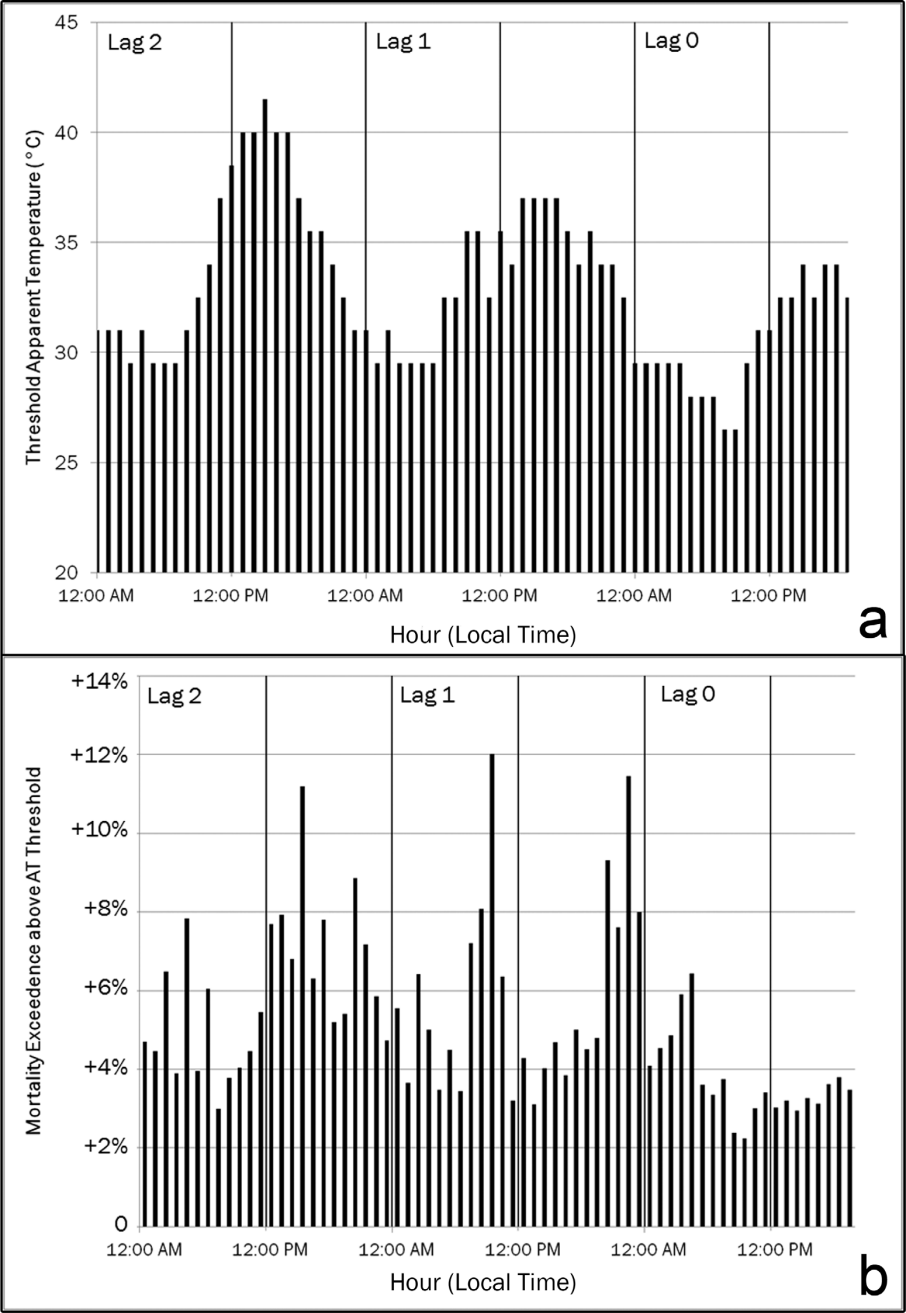
**Hourly threshold apparent temperatures and response for heat-related mortality in Philadelphia County**. (a) Threshold apparent temperatures for heat-related mortality in Philadelphia county, PA. (b) Mortality exceedances for Philadelphia County, PA when apparent temperatures exceed the threshold between midnight and 8:00 p.m. local time on the day of death (lag 0), and for each of the 24 hours on the two days prior.

Countywide mortality is significantly elevated on and following days with high ATs. A significant threshold AT is evident for each hour spanning the 3-day period leading up to and including the day of death (Figure 3a). The threshold temperature varies by hour such that higher afternoon ATs are associated with the same mortality response as lower morning ATs. The mean mortality exceedance when ATs occur above the threshold is 5.2%; however, the cumulative response varies based upon when during the day high ATs occur (Figure 3b). Over the 68-hour period, we observed three peaks in the mortality rate when ATs occurred above the threshold: most of the afternoon hours two days prior to death, the mid-morning hours (7:00 a.m. to 11:00 a.m.) on the day prior to death, and the late afternoon and evening hours (8:00 p.m. to midnight) on the day prior to death.

We focused our local-scale analysis on cases where the 1-day lag 7:00 p.m. local time AT occurred above 34°C. This specific AT/time combination was chosen because of the robustness of the significant elevation in citywide mortality (based on the randomization test for the 35.5°C bin) and the sample size, with 110 such occurrences over the period of record. Citywide mortality increases 9.3% following days with a 7:00 p.m. AT above 34°C (see Figure 3b). Significantly elevated mortality, however, is only observed in 10 of the 48 ZCTAs within Philadelphia County (Figure 1). The remainder of the ZCTAs do not show a significant increase. ZCTAs associated with higher mortality following hot days are located in the central and west portions of the County.

Six principal components were extracted from the pool of 25 variables by analyzing a scree plot (all have eigenvalues ≥ 1.0). Collectively they account for 84.3% of the variability originally present in the dataset (Table 2). PC1 (35.9% explained variance) is highly correlated with surface temperatures from the two satellite images and is also strongly related to socioeconomic status. Component 1 scores are also positive in ZCTAs with an abundance of high density residential housing and high percentages of residents below poverty thresholds and lacking a high school diploma.

**Table 2:** Land use zoning categories aggregated from Philadelphia Zoning Code

Zoning Category	Description
Low Density Residential	Suburban, single-family, detached households with lawns

Mid Density Residential	Semi-detached or attached households with some green space

High Density Residential	Attached households with minimal or no green space

Recreational	Parks and protected natural areas

Industrial	All industrial complexes, stockyards, or ports

Commercial	Center-city office buildings, retails centers, corner shops

Mixed-Use	Strip malls, movie theaters, stadiums, hospitals, colleges, condominiums

**Figure 4:**
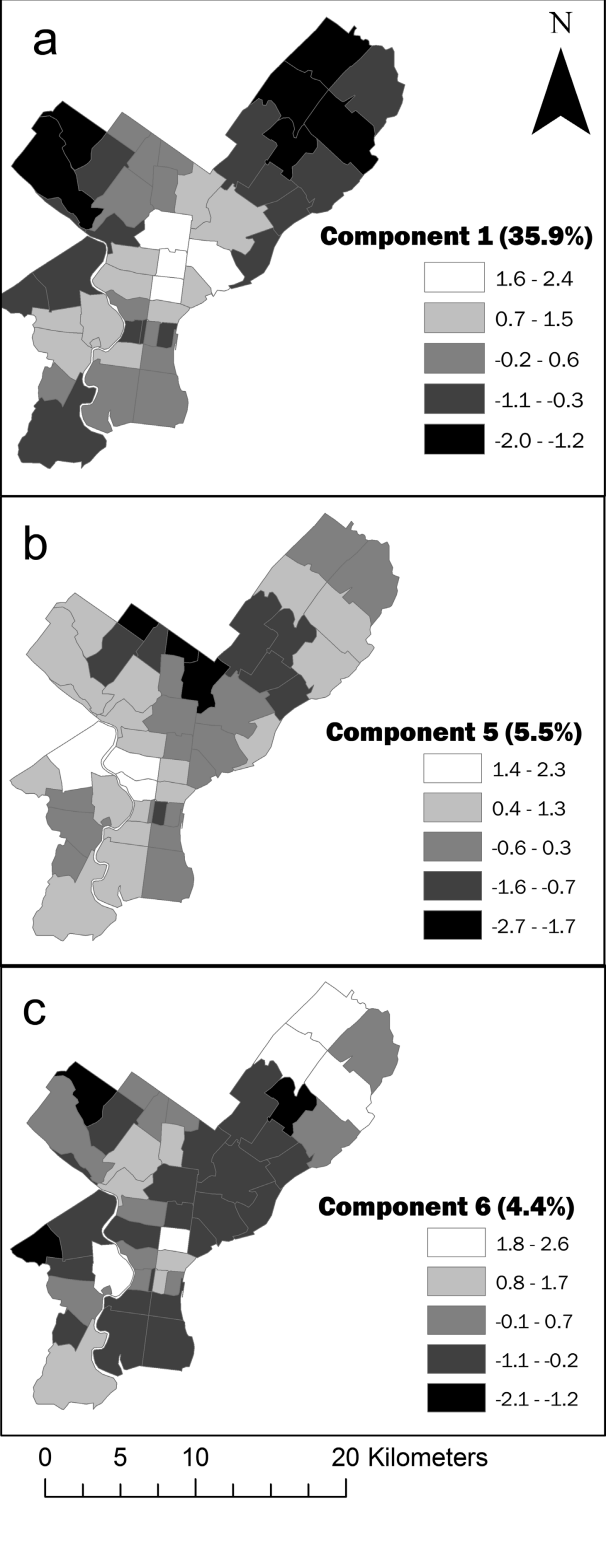
**Principal component scores by ZCTA of significant factors in regression model**. Principal component scores by ZCTA for three significant components (a: PC1, b: PC5, c: PC6) included in regression model relating explanatory variables to heat-related mortality. The percentage of variance explained by each component is shown in parentheses.

Three principal components were significant in the regression model: PC1, PC5, and PC6 (Figure 4). PC5 is representative of high density housing and mixed-use zoning. PC6 is most closely related to age and mixed-use zoning (see Table 1). The regression model identified a significant relationship (*p *< 0.001) between the three components and ZCTA-level mortality

M*=0.019+0.017(PC1)+0.012(PC5)-0.008(PC6)

where M* is the predicted mortality rate (deaths per 10,000 per day when the AT threshold is exceeded) within each zip code, and PC refers to the respective principal components. The partial *p*-values for the coefficients were < 0.001, 0.002, and 0.027, respectively, and the overall model adjusted R^2 ^was 0.439. The model indicates that heat-related mortality is greatest in areas with 1) a high number of residents below poverty thresholds, 2) residents lacking a high school diploma, 3) residents living in high-density housing, 4) more elderly persons, 5) high surface temperatures, and 6) mixed-use zoning.

**Figure 5:**
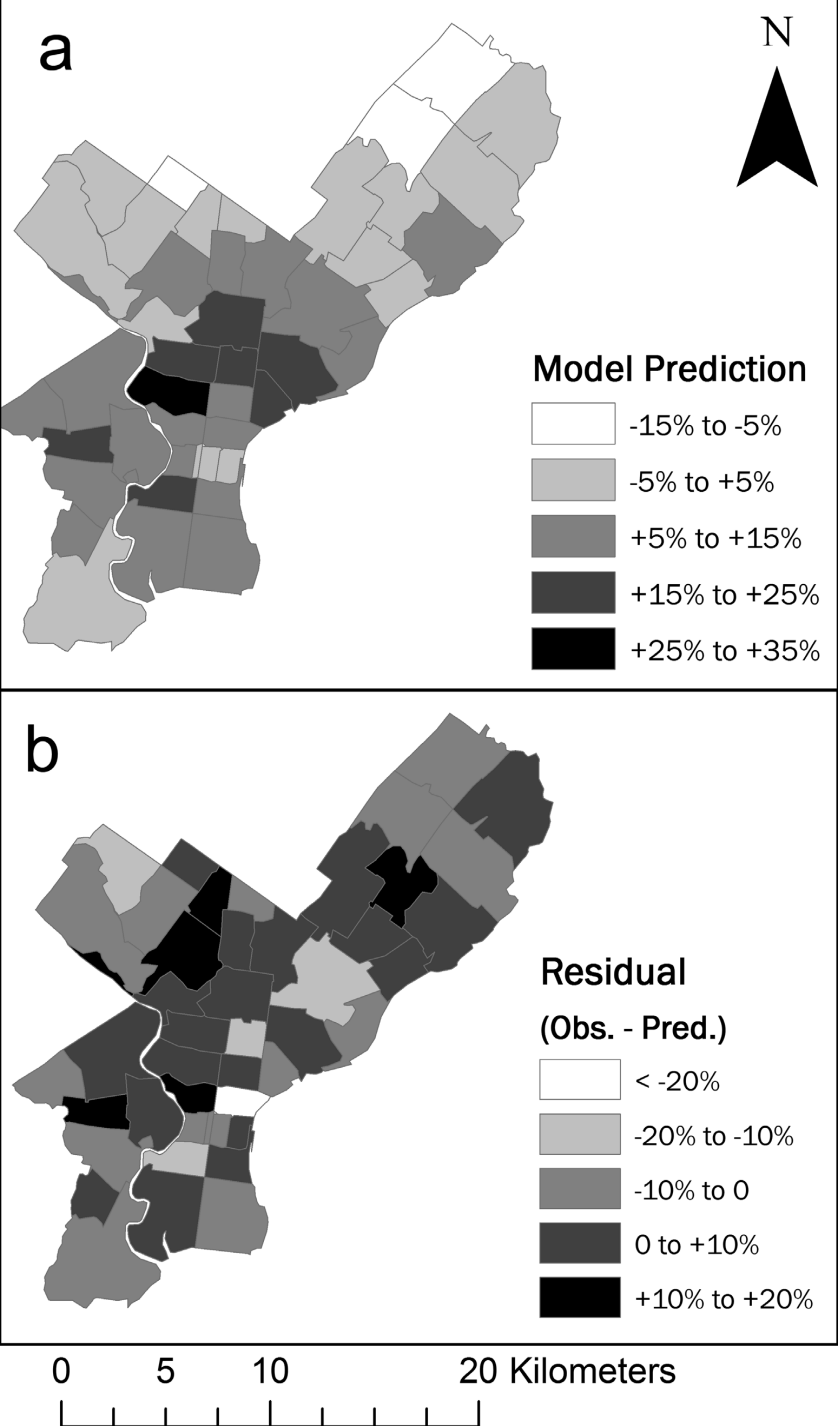
**Regression model predictions and residuals by ZCTA**. (a) Regression model predicted mortality exceedances for Philadelphia County ZCTAs on days following 7:00 p.m. local time apparent temperatures above 34°C. (b) Differences between observed mortality rates and model predictions by ZCTA.

**Figure 6:**
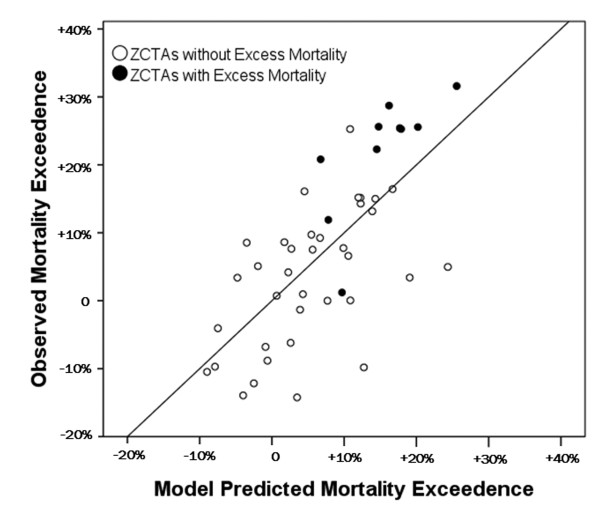
**Comparison of model predictions and observations**. Scatterplot comparing observed mortality rates and model predictions on days following 7:00 p.m. local time apparent temperatures above 34°C by ZCTA. The 1:1 line is included for reference. Filled circles identify the 10 ZCTAs with statistically significant mortality exceedances in Figure 1.

The regression model performs well in identifying high-mortality locations (Figures 5 and 6). Each ZCTA with observed significant mortality exceedances was predicted to have a high mortality rate by the regression model. The range of values predicted by the model is not as great as the range in observed values, and in a few cases, model error is of the same order of magnitude as the observed mortality departures themselves. Residuals from the regression model were found to be randomly spatially distributed as measured by Moran's I statistic (I index 0.08, Z-score 1.13), indicating that an additional term in the model to account for spatial autocorrelation is not needed.

## Discussion

The proper definition of environmental conditions that cause heat-related morbidity and mortality is an unsettled question in human biometeorological research. Various studies have employed different threshold variables, such as maximum temperature, afternoon AT, and morning dew point temperature [7]. Here, we attempt to address this shortcoming by examining AT diurnality for Philadelphia County. Although there are significant mortality elevations when ATs exceed the threshold at any hour, we found three periods with exceedances greater than 10%. Mortality rates were highest when thresholds were exceeded in the morning or evening hours on the day immediately prior to death and in the afternoon two days prior. Examining effects by hour, rather than using more conventional metrics like daily maximum, minimum, or mean temperature allows for a more specific identification of hazardous periods. These patterns may arise in part from the threshold chosen for each hour: the threshold temperature might be expected to follow an even smoother pattern than that shown in Figure 3a. In particular, the threshold temperature seems to increase rapidly on lag 1 between roughly 6:00 and 10:00 a.m., and thus the relatively high values here might be leading to the spike in the response at the same time in Figure 3b. The other peaks in the response curve (Figure 3b) seem less likely to be influenced by variations in the threshold curve. Future work might examine the mortality response above various percentiles of hourly temperature rather than a mortality-based threshold. The lack of an especially high response on the day of death (Figure 3b) may arise from the absence of time for exposure and resultant physiological stress (i.e., the response is not immediate.)

Mortality following days with high ATs in Philadelphia County is not randomly spatially distributed but is concentrated in several distinct regions. Certain ZCTAs exhibit mortality that is more than 30% above the daily citywide average for particular AT-time combinations. Intra-County variability in heat-related mortality has been observed or suggested elsewhere [10,13-18], but the majority of studies to date have focused on a larger spatial scale, single heat events, hot summers, or did not consider the actual mortality response. This study is among the first to quantify local-scale mortality responses over a multi-decadal period.

Several of the variables associated with higher local-scale mortality are consistent with observations and hypotheses in the literature, including high-density housing, low socioeconomic status, high surface temperatures, and elderly populations [13,16]. The spatial distribution of heat-related mortality in Philadelphia County during the 1993 heat wave was previously examined and the same variables were associated with elevated risk [10,17]. The lack of a strong relationship with recreational zoning is surprising because we expected places with more parks and green space to have lower surface temperatures, thereby reducing heat and heat-related mortality. Recreational zoning is highest in two ZCTAs along the Schuylkill River in the western portion of the County, one of which also has a high percentage of high density residential zoning. However, the two zoning types are not interspersed, and where green space is not intermingled amongst residential areas, the mitigating effect on temperature in dense residential areas may be diminished. Although a large body of research points to the advantages of adding green space to lower temperatures in the urban environment, we are not able to conclude that ZCTAs with more parkland are associated with lower mortality rates. This does not indicate that green space is not beneficial, but rather that many other variables may confound the signal, especially at the scale of this analysis. We are continuing to investigate the relationship between zoning types, air and surface temperatures, and mortality outcomes.

This study also incorporates the relatively recent approach of including remotely-sensed measurements of surface temperature in the study of heat-related mortality. Individuals living in areas with higher surface temperatures are at greater risk following hot days. This finding is consistent with the expectation that individuals living in hotter places are under greater physiological stress [10]. We are encouraged that the results from a remote sensing approach are similar to those using other sensors or models of the UHI.

We did not directly identify race as a key factor in the spatial distribution of heat-related deaths. Principal component loadings for the racial variables were only high in one significant component (PC 1), but loadings for other variables (income, surface temperature, educational attainment, and density of development) were higher. As previously documented for Phoenix [5], minority populations in Philadelphia County live in areas that are associated with higher surface and air temperatures. We directly observed the relationship with surface temperature and can infer the relationship with air temperature because of the high density of residential development in these locales. Racial variable loadings are very small for the other two components included in the model (PCs 5 and 6). Thus, we cannot conclude that race alone is a key factor in the spatial distribution of heat-related deaths in Philadelphia.

There are a few limitations we faced in creating our model for Philadelphia that merit discussion. The sociodemographic and zoning variables were derived from data available at a fixed point in time (e.g., the year 2000 census). However, the underlying demographics and zoning ordinances both change over time, a process we were unable to capture using this approach. This introduces some uncertainty into the results, and future research should explore local-scale mortality patterns over both space and time.

We were especially interested in exploring the relationship between the complex temperature patterns present in the metropolitan area and heat-related mortality. Satellite imagery has become much more accessible and makes this type of analysis possible using surface temperature measurements. The surface temperature field may be much different from the air temperature field over the same area, and we are not claiming that the two are identical, although some research indicates a high degree of spatial correlation between the two fields during daylight hours [24]. There are many aspects of the urban heat island worthy of consideration in the context of urban health, including day/night variability and the contrast between the surface heat island and that of the canopy layer. We are investigating if residents in places with higher morning surface temperatures on hot days are at greater risk.

We are currently implementing a cloud-masking scheme that will increase the number of available images and extending our sample prior to 2004. We believe that the use of remote imagery in our study, and others, could be greatly enhanced if more surface temperature images were used. In just the two used in our study, there is variability in the surface temperature pattern that may be linked to seasonal differences, synoptic-scale conditions, or other environmental controls. An additional concern with the satellite imagery is that many of the image pixels are measuring rooftop temperature, which may not be representative of the surface conditions experienced where individuals might be living, working, or spending time outdoors.

We observed a high correlation between the surface temperature field and several socioeconomic variables, as evidenced by the high loadings on the first principal component. Although principal components allows for the examination of potential effects of a large suite of variables believed to be associated with risk, one tradeoff can be difficulty in interpreting the results. We can definitively say that places with higher surface temperatures are associated with higher mortality risk, but those places also have a high percentage of residents living in poverty and a high percentage of residents without a high school diploma. This pattern has been observed for other cities in the United States [5] and makes it difficult to pinpoint a causal relationship between the individual predictor variables and the health response. Even if it is difficult to separate the effects of individual variables, identifying characteristics of places associated with higher heat-related mortality can lead to improvements in the allocation of medical resources during dangerous conditions. Our future analysis in other cities in the United States where socioeconomic status and surface temperatures may not be as highly correlated may shed light on the relative impact of exposure, education, and income on heat-related risk.

The role of air quality in leading to increased mortality during heat waves is a topic of continued debate in the literature and is beyond the scope of this study. As heat waves are commonly associated with clear skies and stagnant air, conditions are ideal for the rapid buildup and accumulation of various pollutants. It is likely that during heat waves a portion of the excess deaths are attributable to the thermal stress whereas others might be linked to high concentrations of unhealthy atmospheric constituents. We did not incorporate air quality data into this study but encourage future study of the interactive effects of heat and air quality on summertime mortality as well as the potential for differential mortality over space as a result of local-scale air quality variability. Both are topics of active ongoing investigation by the authors and many others. We also note that we were unable to locate air conditioning use data at an appropriate resolution for this study. Air conditioning has become widely adopted in the United States and increases in availability have been linked to decreases in heat-related mortality [2]. However, we believe that air conditioning availability and usage is likely highly correlated with measures of socioeconomic status, and thus may be implicitly included in our analysis. Access and willingness to use medical care is a potential confounder at the individual level that we were not able to represent at the scale of this study, although it may be highly correlated with the socioeconomic variables included. Finally, the use of AT may not identify all of the critical physiological factors in evaluating the heat-mortality relationship and we intend to adopt this approach with other physiological indices in the future.

Intra-city variability in the response to high heat and humidity conditions indicates an opportunity for the improvement of heat-health watch-warning systems (HHWWS) that have been deployed in cities across the globe. When a dangerous event is forecast, for example, emergency managers might reprioritize allocation of medical resources to those geographic areas responsible for the largest portion of the heat-related deaths in the past. A more thorough effort to build and validate a predictive model of both the timing and placement of heat-related deaths is recommended prior to operational changes in any HHWWS. Longer-term strategies to reduce the heat stress and health burden in these localities might be considered as well, such as the implementation of building weatherization programs, adding green space to the city landscape, adoption of low-albedo and/or green building practices, and location of future healthcare facilities.

## Conclusions

We have identified statistically significant fine-scale spatial variability in heat-related mortality within Philadelphia County, PA, over the period 1983-2008. Following days on which the 7:00 p.m. AT exceeds 34°C Countywide, mortality is significantly elevated, but the excess deaths are not randomly distributed throughout the metropolitan area. Instead, only 10 of the 48 individual ZCTAs that comprise the County exhibit significantly higher mortality. Compared with areas that do not have elevated mortality following hot days, these 10 ZCTAs have a higher percentage of elderly residents, a higher percentage of residents of low socioeconomic status, more high-density residential and mixed-use zoning, higher surface temperatures, and more recreationally-zoned area. A portion of the spatial distribution of heat-related mortality arises from underlying demographic, social, and environmental variability. The overall Countywide response varies based on the specific timing and intensity of high heat and humidity. Afternoon AT thresholds are higher than morning thresholds, but especially high mortality rates are observed when the threshold is crossed either during the mid-morning or late afternoon hours.

The significant local-scale variability in heat-related mortality identified for Philadelphia County suggests an opportunity for improved heat preparedness and management strategies. In the case of alerting the public, localities associated with excess mortality could receive additional notification or special forecasts when hot conditions are expected. These places are also prime candidates for facilities that can help residents escape the impact of high ATs. The authors are adopting this approach for other United States cities in different climate zones to determine if certain factors are consistently associated with elevated risk during heat waves. Understanding the characteristics of places especially sensitive to the dangers of high heat and humidity may ultimately reduce the impact of extreme summertime conditions on human health.

## Abbreviations

AT: Apparent temperature; ZCTA: Zip Code Tabulation Area.

## Competing interests

The authors declare that they have no competing interests.

## Authors' contributions

DH is responsible for the overall study design, completed all processing of mortality data, performed the statistical analysis for spatial modeling, and drafted the manuscript. RD contributed to study design, coordination, and helped draft the manuscript. ML acquired and prepared the zoning data using GIS. MS was responsible for obtaining the climatic data and constructed much of the code for threshold analysis. CW acquired and processed remote sensing imagery. LV obtained and prepared the sociodemographic data using GIS. All authors read and approved the final manuscript.
